# Multidomain Assessment of the Association Between Oral and Cognitive Function in Community-Dwelling Older Adults: A Cross-Sectional Study

**DOI:** 10.7759/cureus.109566

**Published:** 2026-05-24

**Authors:** Ikue Kondo, Atsunori Itagaki, Yugo Kimura, Shihoko Yoshida, Takumi Saito, Issei Sugimoto, Tomohito Nunomura, Sumika Ogawa, Sangun Lee

**Affiliations:** 1 Health Sciences, Graduate School of Health Sciences, Aomori University of Health and Welfare, Aomori, JPN; 2 Speech, Language and Hearing Therapy, Mejiro University, Tokyo, JPN; 3 Physical Therapy, Tokyo Metropolitan University, Arakawa, JPN; 4 Rehabilitation, National Hospital Organization Kamaishi Hospital, Kamaishi, JPN; 5 Physical Therapy, Aomori University of Health and Welfare, Aomori, JPN; 6 Rehabilitation, Matsuda Hospital, Sendai, JPN; 7 Rehabilitation, Aomori Prefectural Central Hospital, Aomori, JPN

**Keywords:** aged, community-dwelling older adults, japanese geriatrics, mild cognitive impairment (mci), oral health, speech production measurement, tongue

## Abstract

Aim: Oral function is considered a potentially modifiable factor associated with cognitive decline; however, few studies have evaluated multiple domains of oral function to determine which measures are independently associated with mild cognitive impairment (MCI) in community-dwelling older adults.

Methods: This cross-sectional study included community-dwelling older adults aged ≥ 60 years in a rural city in Niigata Prefecture, Japan. Cognitive function was evaluated using the Japanese version of the Montreal Cognitive Assessment (MoCA-J), with scores < 25 indicating MCI. Six oral function measures, based on diagnostic criteria for oral hypofunction, were assessed: tongue coating index (TCI), oral moisture, masticatory function (a* value), swallowing function (Oral Frailty 5-item Checklist, OF-5), oral diadochokinesis (ODK) /ta/, and maximum tongue pressure. Correlation and logistic regression analyses were performed, and statistical significance was defined as p < 0.05.

Results: Among 303 participants, 179 had MCI. In the fully adjusted model, ODK /ta/ (OR = 0.621, p = 0.012), maximum tongue pressure (OR = 0.959, p = 0.014), and masticatory function (OR = 0.945, p = 0.044) were independently and inversely associated with MCI. Oral moisture, TCI, and OF-5 were not significantly associated. ODK /ta/ showed the strongest correlation with MoCA-J scores (r = 0.302, p < 0.001).

Conclusions: ODK /ta/, maximum tongue pressure, and masticatory function were independently associated with MCI in community-dwelling older adults. Among these, ODK /ta/ showed the strongest correlation with MoCA-J scores (r = 0.302). These findings suggest that multidomain assessment of oral function may serve as a useful indicator for identifying older adults at risk of cognitive decline, warranting further longitudinal investigation.

## Introduction

The global expansion of the older adult population has been accompanied by a marked increase in dementia prevalence. Approximately 40% of dementia cases may be preventable or delayed through modification of risk factors [1], underscoring the importance of early detection and prevention. Mild cognitive impairment (MCI) is a transitional stage preceding dementia, with an annual conversion rate of 5-15% [2]. However, MCI may represent a reversible phase during which appropriate interventions can restore normal cognitive function [3]. Therefore, early identification and intervention at the MCI stage are critical strategies for dementia prevention among older adults [1]. Such interventions may include oral function training, nutritional counseling, and exercise programs targeting modifiable risk factors.

Increasing attention has focused on the association between declining oral function and cognitive impairment. Oral function decline has been linked to adverse outcomes, including increased mortality, physical frailty, and reduced quality of life [4]. Specifically, mastication may enhance hippocampal blood flow and neuronal activity, whereas a decline in oral function may impair nutritional intake through reduced chewing efficiency, thereby indirectly affecting cognitive processes [5]. Previous cross-sectional and longitudinal studies have demonstrated associations between tongue pressure, oral diadochokinesis (ODK), masticatory function, and MCI [6-12]. Notably, tongue pressure and ODK have been independently associated with cognitive function in studies evaluating multiple oral function domains simultaneously [6,7].

Despite these findings, few studies have assessed multiple domains of oral function using the diagnostic criteria for oral hypofunction while examining the independent contribution of each domain to MCI within a single cohort. Furthermore, the relative strength of these associations and the independence of each measure remain unclear. The present study evaluated six oral function domains in community-dwelling older adults and examined their independent associations with MCI, aiming to provide evidence to support early detection of cognitive decline.

## Materials and methods

Methods

Participants

This study was coordinated through the Graduate School of Health Sciences, Aomori University of Health and Welfare, Aomori, Japan, and conducted as part of a community health survey in collaboration with the local government. Community-dwelling older adults aged ≥ 60 years residing in a rural city in Niigata Prefecture, Japan, were recruited voluntarily. Participants were recruited using a convenience sampling approach based on the availability of community members for voluntary participation during the survey period. No formal a priori sample size calculation was performed; however, the adequacy of the final sample was evaluated post hoc using the events-per-variable (EPV) criterion. With 179 MCI cases and 11 covariates included in the logistic regression models, the EPV was approximately 16, exceeding the commonly recommended minimum value of 10 [13]. All participants provided written informed consent prior to participation.

**Figure 1:**
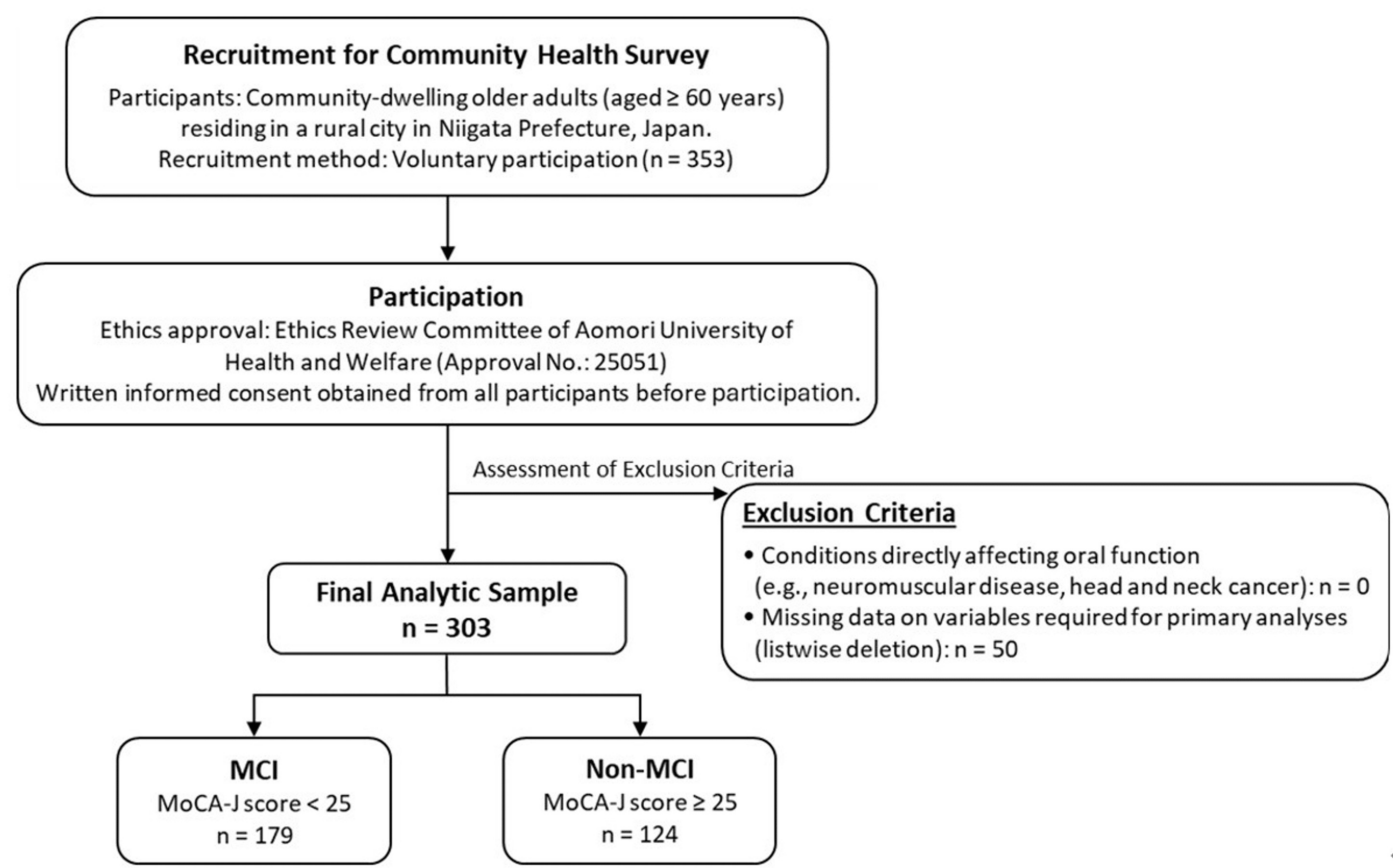
Flowchart of the participant selection process Image created by authors using PowerPoint (Microsoft Corporation, Redmond, WA). MCI: mild cognitive impairment; MoCA-J: Japanese version of the Montreal Cognitive Assessment

Inclusion criteria were community-dwelling status and age ≥ 60 years. Exclusion criteria included conditions directly affecting oral function, such as neuromuscular disease, head and neck cancer, or other relevant medical conditions. Participants with missing data for variables required for the primary analyses were also excluded through listwise deletion, resulting in a final analytic sample of 303 participants (Figure 1).

All oral function assessments were conducted by licensed healthcare professionals, including physical therapists, occupational therapists, speech-language-hearing therapists, nurses, and dental hygienists with relevant clinical expertise. Trained volunteers provided supplementary assistance, including participant guidance, data recording, and disinfection of measurement equipment, but did not perform any clinical assessments.

This study was conducted with approval from the Ethics Review Committee of Aomori University of Health and Welfare (approval number: 25051).

Measures

Demographic data were collected, including age, sex, years of education, lifestyle factors (smoking history, alcohol consumption, and physical activity), and medical history. Physical activity was assessed using the International Physical Activity Questionnaire (IPAQ), with ≥ 600 METs·min/week defined as regular exercise [8]. Comorbidity burden was evaluated using the modified Charlson Comorbidity Index (modified CCI).

Cognitive function assessment

Cognitive function was assessed using the Japanese version of the Montreal Cognitive Assessment (MoCA-J), which evaluates seven domains: visuospatial/executive function, naming, memory, attention, language, abstraction, and orientation. Total scores range from 0 to 30. Participants with scores < 25 were classified as having MCI, whereas those with scores ≥25 were classified as non-MCI [14].

Oral function assessment

Oral function was assessed across six domains based on the diagnostic criteria for oral hypofunction [15]. Although the diagnostic criteria include seven domains - oral hygiene, oral dryness, masticatory function, swallowing function, articulatory function, tongue pressure, and oral sensation - oral sensation was excluded because its assessment relies primarily on subjective methods, and no standardized objective measurement tool has been established for use in large-scale community surveys. The remaining six domains were evaluated using an objective measure. Oral hygiene was evaluated using the Tongue Coating Index (TCI), with ≥ 50% indicating poor status [16].

Oral dryness was measured using an oral moisture-checking device (Mucus®, Yoshida Co., Ltd., Tokyo, Japan), with values < 27 indicating dryness. TCI and oral moisture were not assessed in all participants due to equipment availability constraints during the community survey (TCI: n = 203; oral moisture: n = 237).

Masticatory function was assessed using xylitol chewing gum (Lotte Co., Ltd., Tokyo, Japan), with the a* value quantified using a colorimeter (CR-10Plus/CR-20, Konica Minolta, Inc., Tokyo, Japan) [17]. Higher a* values indicate better masticatory performance. Swallowing function was evaluated using the Oral Frailty 5-item Checklist (OF-5), with ≥ 2 endorsed items indicating oral frailty [18].

Articulatory function was measured using ODK /ta/ with the Kenkou-kun Handy II device (Takei Scientific Instruments Co., Ltd., Tokyo, Japan). ODK /ta/ was selected based on the study protocol because of the time constraints inherent in community surveys and because /ta/, which reflects tongue tip movement, has been reported to be useful for the early evaluation of oral function decline in older adults [19]. Future studies should examine whether /pa/ and /ka/ show similar associations with MCI. Maximum tongue pressure was measured using the JMS Tongue Pressure Measurement Device (TPM-01, JMS Co., Ltd., Kyoto, Japan), with the higher value from two trials recorded [20].

Statistical analysis

Statistical analyses were performed using Statistical Product and Service Solutions (SPSS, version 31.0; IBM SPSS Statistics for Windows, Armonk, NY). Between-group comparisons were conducted using the Mann-Whitney U and chi-square tests. Spearman's correlation coefficients were calculated to assess associations between MoCA-J scores and continuous oral function measures (oral dryness, masticatory function, ODK /ta/, and maximum tongue pressure).

Binomial logistic regression analysis was conducted to examine independent associations, adjusting for age, sex, years of education, BMI, dyslipidemia, modified CCI, regular exercise, and living status. ODK /ta/, maximum tongue pressure, and masticatory function were entered simultaneously into a combined model because complete data were available for all 303 participants and because these measures showed significant correlations with MoCA-J scores in the Spearman correlation analyses. TCI, oral moisture, and OF-5 were analyzed in separate individual models because of incomplete data availability for TCI and oral moisture (TCI: n = 203; oral moisture: n = 237) and because none of these three measures showed a significant correlation with MoCA-J scores, suggesting that they may operate through pathways distinct from those of the variables included in the combined model.

To assess potential selection bias introduced through listwise deletion, basic characteristics (age and sex) of excluded participants (n = 50) were compared with those of participants included in the final analytic sample (n = 303) using the Mann-Whitney U test and chi-squared test. Similarly, to examine whether missing data for TCI and oral moisture were associated with participant characteristics, age, sex, and MoCA-J scores were compared between participants with and without these measurements using the same tests. Statistical significance was defined as p < 0.05.

## Results

Participants

Participants' characteristics are presented in Table 1. Among 303 participants, 179 (59.1%) were classified as having MCI and 124 (40.9%) as non-MCI. The MCI group was significantly older (p < 0.001) and had fewer years of education (p = 0.033) than the non-MCI group. The prevalence of dyslipidemia was lower in the MCI group (p = 0.009).

**Table 1:** Baseline characteristics of the participants MCI: mild cognitive impairment, BMI: body mass index, Modified CCI: Modified Charlson Comorbidity Index ^1^Continuous variables are expressed as median (IQR): Mann-Whitney U test. Categorical variables are expressed as n (%): chi-square test.

Variable	Total (n=303)	Non-MCI (n=124)	MCI (n=179)	p-value
Age, years ¹	73.0 (69-77)	70.5 (66-75)	75.0 (71-80)	<0.001
Education, years ¹	12 (12-14)	12 (1214)	12 (12-12)	0.033
Body mass index (BMI) ¹	23.0 (21.1-25.2)	23.0 (21.2-25.5)	23.0 (20.6-25.1)	0.454
Sex (Male), n (%)	71 (23.4)	24 (19.4)	47 (26.3)	0.163
Hypertension, n (%)	121 (39.9)	51 (41.1)	70 (39.1)	0.724
Diabetes mellitus, n (%)	25 (8.3)	12 (9.7)	13 (7.3)	0.453
Dyslipidemia, n (%)	59 (19.5)	33 (26.6)	26 (14.5)	0.009
Cancer history, n (%)	40 (13.2)	16 (12.9)	24 (13.4)	0.898
Modified CCI ¹	1 (0-1)	1 (0-1)	1 (0-1)	0.572
Living alone, n (%)	47 (15.5)	18 (14.5)	29 (16.2)	0.690
Regular exercise, n (%)	120 (39.6)	53 (42.7)	67 (37.4)	0.353

To assess potential selection bias, the basic characteristics of excluded participants (n = 50) were compared with those of participants included in the final analytic sample. No significant difference was observed in sex distribution (chi-squared test, p = 0.609); however, excluded participants were significantly older than included participants (Mann-Whitney U test, p = 0.012).

Oral function

Descriptive statistics and between-group comparisons for the six oral function measures are shown in Table 2. The MCI group exhibited lower masticatory function (median a* value: 19.6 vs. 20.3, p = 0.029), ODK /ta/ (6.1 vs. 6.5 repetitions/s, p < 0.001), and maximum tongue pressure (32.2 vs. 36.4 kPa, p < 0.001). No significant between-group differences were observed for oral moisture, TCI, or OF-5.

**Table 2:** Oral function measures by mild cognitive impairment (MCI) status ^1^Continuous variables are expressed as medians (IQRs): Mann-Whitney U test. Categorical variables are expressed as n (%): chi-square test. ^2^TCI was assessed in a subset of participants (n = 203); oral moisture was assessed in a subset (n = 237). All other measures were available for all 303 participants. TCI: Tongue Coating Index; OF-5: Oral Frailty 5-item Checklist; ODK: oral diadochokinesis

Oral function measure	Total (n=303)	Non-MCI (n=124)	MCI (n=179)	p-value
Articulatory function (ODK /ta/) ¹	6.4 (5.9-6.9)	6.5 (6.0-6.8)	6.1 (5.6-7.0)	< 0.001
Maximum tongue pressure, kPa ¹	33.8 (28.1-39.5)	36.4 (29.7-42.7)	32.2 (27.1-38.4)	< 0.001
Masticatory function (a* value)¹	19.9 (17.0-22.8)	20.3 (16.9-23.3)	19.6 (17.4-23.6)	0.029
Oral dryness ¹	29.8 (28.1-31.4)	29.3 (27.5-31.2)	29.9 (28.7-31.9)	0.202
Oral hygiene (TCI ≥50%), n (%)	44/203 (21.7)	21/103 (20.4)	23/100 (23.0)	0.652
Swallowing function (OF-5), n (%)	48 (15.8)	20 (16.1)	28 (15.6)	0.909

To examine whether missing data for TCI and oral moisture were associated with participant characteristics, comparisons were conducted between participants with and without these measurements. For TCI, no significant differences were observed in age (p = 0.077) or sex (p = 0.062); however, participants with missing TCI data had significantly lower MoCA-J scores than those with available data (p < 0.001). Similarly, for oral moisture, no significant differences were observed in age (p = 0.832) or sex (p = 0.405); however, participants with missing oral moisture data had significantly lower MoCA-J scores (p = 0.023).

Correlation analyses

Spearman's correlation coefficients between MoCA-J scores and oral function measures are shown in Table 3. ODK /ta/ (r = 0.302, p < 0.001), maximum tongue pressure (r = 0.267, p < 0.001), and masticatory function (r = 0.165, p = 0.004) were positively correlated with MoCA-J scores, with ODK /ta/ showing the strongest association. Oral moisture was not significantly correlated with MoCA-J scores (r = -0.054, p = 0.404).

**Table 3:** Spearman correlations between MoCA-J scores and oral function measures MoCA-J: Japanese version of the Montreal Cognitive Assessment

Oral function measure	Spearman's rho	p-value
Articulatory function: ODK /ta/ (reps/s)	0.302	< 0.001
Maximum tongue pressure (kPa)	0.267	< 0.001
Masticatory function (a* value)	0.165	0.004
Oral dryness (moisture value)	-0.054	0.404

Logistic regression analysis

**Figure 2:**
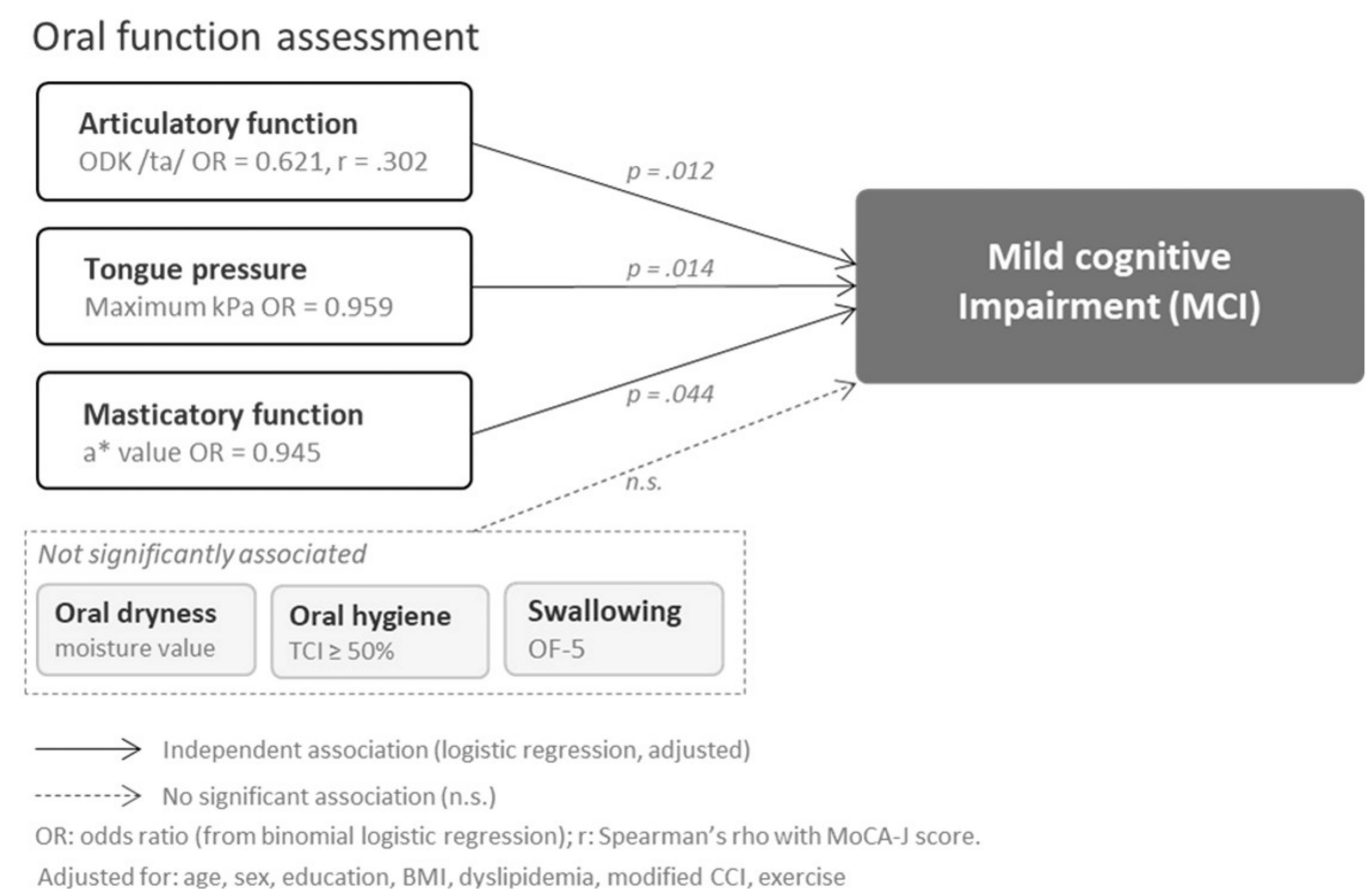
Schematic of independent associations between oral function measures and mild cognitive impairment Solid arrows indicate significant independent associations based on binomial logistic regression (fully adjusted). Dashed arrows indicate non-significant associations. OR: odds ratio; r: Spearman's rho with MoCA-J score. Adjusted for: age, sex, years of education, BMI, dyslipidemia, modified CCI, and regular exercise. MCI: mild cognitive impairment; MoCA-J: Montreal Cognitive Assessment, Japanese version; ODK: oral diadochokinesis; TCI: Tongue Coating Index; OF-5: Oral Frailty 5-item Checklist; Modified CCI: Modified Charlson Comorbidity Index. Created using PowerPoint (Microsoft Corporation, Redmond, WA)

Results of the binomial logistic regression analysis are presented in Table 4. MCI status (MoCA-J < 25) was the dependent variable, with age, sex, years of education, BMI, dyslipidemia, modified CCI, regular exercise, and living status included as covariates. In the combined model, ODK /ta/ (OR = 0.621, 95% CI: 0.427-0.901, p = 0.012), maximum tongue pressure (OR = 0.959, 95% CI: 0.927-0.991, p = 0.014), and masticatory function (OR = 0.945, 95% CI: 0.895-0.998, p = 0.044) were independently and inversely associated with MCI; higher values corresponded to lower odds of MCI. Age remained a strong predictor across models (OR = 1.096, 95% CI: 1.043-1.151, p < 0.001). In individual models, TCI (OR = 1.180, 95% CI: 0.561-2.483, p = 0.663), oral moisture (OR = 1.038, 95% CI: 0.952-1.132, p = 0.394), and OF-5 (OR = 1.011, 95% CI: 0.505-2.025, p = 0.974) were not significantly associated with MCI. The independent associations between oral function measures and MCI are summarized in Figure 2.

**Table 4:** Logistic regression analysis of the association between oral function measures and mild cognitive impairment (MCI) All models adjusted for age, sex, years of education, BMI, dyslipidemia, modified Charlson Comorbidity Index, regular exercise, and living alone. Individual models: TCI (n = 203), oral moisture (n = 237), and OF-5 (n = 303) were analyzed separately. OR: odds ratio; CI: confidence interval; MCI: mild cognitive impairment; TCI: Tongue Coating Index; OF-5: Oral Frailty 5-item Checklist; ODK: oral diadochokinesis Combined model: ODK /ta/, maximum tongue pressure, and masticatory function (a* value) entered simultaneously (n = 303).

Oral function measure	n	OR	95% CI	p-value
Combined model (ODK /ta/ + tongue pressure + masticatory function)				
Articulatory function: ODK /ta/ (reps/s)	303	0.621	0.427-0.901	0.012
Maximum tongue pressure (kPa)	303	0.959	0.927-0.991	0.014
Masticatory function (a* value)	303	0.945	0.895-0.998	0.044
Individual models				
Oral hygiene: TCI ≥50%	203	1.180	0.561-2.483	0.663
Oral dryness (moisture value)	237	1.038	0.952-1.132	0.394
Swallowing function: OF-5 positive	303	1.011	0.505-2.025	0.974

## Discussion

This study evaluated six oral function domains based on diagnostic criteria for oral hypofunction in community-dwelling older adults and examined their associations with MCI. After adjustment for demographic and health-related factors, ODK /ta/, maximum tongue pressure, and masticatory function were independently associated with MCI, whereas oral dryness, oral hygiene, and swallowing function were not.

ODK /ta/ showed the strongest correlation with MoCA-J scores (r = 0.302) and was independently associated with MCI in the multivariate analysis (OR = 0.621). ODK /ta/ specifically assesses repetitive movements of the tongue tip and anterior tongue, which may be particularly sensitive to subtle motor decline associated with cognitive changes. Prior studies have reported that ODK, including /ta/, is associated with cognitive function [6,8] and useful for the early evaluation of oral function decline in older adults [18]. Although only /ta/ was assessed in the present study due to protocol constraints, future studies should examine whether /pa/ and /ka/ show similar or distinct associations with MCI. Kugimiya et al. reported that reduced tongue pressure was indirectly associated with lower Mini-Mental State Examination (MMSE) scores via ODK [6], suggesting that ODK may integrate multiple oral motor functions relevant to cognition. Similarly, Mishima et al. found that ODK, specifically /pa/, was the only oral function measure independently associated with MCI [9], indicating that articulatory motor function may be broadly related to cognitive status. Longitudinal evidence further suggests that a decline in oral function, including ODK, is associated with increased risk of dementia onset [12], supporting its role as a potential early indicator of cognitive decline.

Maximum tongue pressure was also independently associated with MCI after adjustment. This finding is consistent with Nakamura et al. [7], who reported an independent association between reduced tongue pressure and MCI (OR = 1.77). Nagatani et al. [11] demonstrated that oral frailty, including reduced tongue pressure, predicted incident MCI in longitudinal analysis. The persistence of this association after adjusting for age indicates that it is not solely attributable to aging. The lack of a significant association with OF-5 suggests that tongue pressure may relate to cognitive function through mechanisms distinct from swallowing function.

Reduced masticatory function, assessed by the a* value of xylitol chewing gum, was also independently associated with MCI. Prior studies using objective measures have reported similar associations between impaired mastication and cognitive decline [21]. Mastication influences nutritional intake and may enhance hippocampal blood flow and neuronal activity [5], providing a plausible biological mechanism. These findings align with Kugimiya et al. [6], who observed a significant correlation between masticatory function and MMSE scores. Because masticatory function is modifiable through interventions such as denture adjustment or masticatory training, it represents a practical target for prevention strategies. Collectively, ODK /ta/, maximum tongue pressure, and masticatory function may share underlying neural pathways linking oral and cognitive function.

In contrast, oral dryness, TCI, and OF-5 were not associated with MCI. These measures may be more influenced by subjective reporting or lifestyle factors and may be less sensitive to cognitive-related changes than objective measures, such as tongue pressure, ODK, and masticatory function. Previous studies have reported inconsistent findings regarding swallowing function and oral hygiene [7,9], which is consistent with the present results.

A key strength of this study is the multidomain evaluation of oral function within a single cohort using standardized criteria. The use of multivariate analysis enabled the assessment of independent associations and comparison of their relative strength. Future research should prioritize longitudinal designs to establish temporal and causal relationships between oral function decline and MCI onset. Development of composite screening tools integrating ODK, tongue pressure, and masticatory function - potentially alongside cognitive screening instruments - represents a promising avenue for clinical translation.

This study has several limitations. First, the cross-sectional design precludes causal inference. Both directions of association are plausible: oral function decline may contribute to MCI, or MCI may impair oral self-management. Second, participants were community-dwelling older adults recruited voluntarily, which may introduce selection bias toward health-conscious individuals. Longitudinal studies are required to clarify temporal relationships. Third, several potential confounders were not assessed, including the number of remaining teeth, denture use, nutritional status, depressive symptoms, frailty status, medication use, and socioeconomic factors, all of which may independently influence both oral function and cognitive status. Finally, TCI and oral moisture were not assessed in all participants due to logistical constraints across multiple survey sessions. Participants with missing TCI or oral moisture data had significantly lower MoCA-J scores than those with available data (TCI: p < 0.001; oral moisture: p = 0.023), suggesting non-random missingness that may have introduced selection bias. Furthermore, participants excluded from the final analytic sample because of missing data for other variables (n = 50) were significantly older than included participants (p = 0.012), potentially reflecting greater difficulty completing questionnaires or performing certain assessments among older individuals. These findings suggest that the results for TCI and oral moisture, as well as the generalizability of the overall findings to older adults with incomplete data, should be interpreted with caution.

## Conclusions

Multidomain assessment of six oral function domains in community-dwelling older adults demonstrated that ODK /ta/, maximum tongue pressure, and masticatory function were independently associated with MCI after adjustment for age and comorbidities, with ODK /ta/ showing the strongest correlation with MoCA-J scores. These findings suggest the potential utility of these measures as indicators for identifying older adults at risk of cognitive decline. In contrast, oral dryness, oral hygiene, and swallowing function were not associated with MCI. Longitudinal studies are warranted to establish causal relationships, and the potential utility of combined oral function assessments as preventive interventions for cognitive decline warrants further investigation.

## References

[1] Livingston G, Huntley J, Sommerlad A (2020). Dementia prevention, intervention, and care: 2020 report of the Lancet Commission. Lancet.

[2] Mitchell AJ, Shiri-Feshki M (2009). Rate of progression of mild cognitive impairment to dementia - meta-analysis of 41 robust inception cohort studies. Acta Psychiatr Scand.

[3] Canevelli M, Grande G, Lacorte E (2016). Spontaneous reversion of mild cognitive impairment to normal cognition: a systematic review of literature and meta-analysis. J Am Med Dir Assoc.

[4] Dibello V, Lobbezoo F, Lozupone M (2023). Oral frailty indicators to target major adverse health-related outcomes in older age: a systematic review. Geroscience.

[5] Weijenberg RA, Delwel S, Ho BV, van der Maarel-Wierink CD, Lobbezoo F (2019). Mind your teeth - the relationship between mastication and cognition. Gerodontology.

[6] Kugimiya Y, Ueda T, Watanabe Y, Takano T, Edahiro A, Awata S, Sakurai K (2019). Relationship between mild cognitive decline and oral motor functions in metropolitan community-dwelling older Japanese: the Takashimadaira study. Arch Gerontol Geriatr.

[7] Nakamura M, Hamada T, Tanaka A (2021). Association of oral hypofunction with frailty, sarcopenia, and mild cognitive impairment: a cross-sectional study of community-dwelling Japanese older adults. J Clin Med.

[8] (2026). World Health Organization: WHO guidelines on physical activity and sedentary behaviour. https://www.who.int/publications/i/item/9789240015128.

[9] Mishima Y, Nakamura M, Matsuda Y (2025). Association between cognitive impairment and poor oral function in community-dwelling older people: a cross-sectional study. Healthcare (Basel).

[10] Egashira R, Mizutani S, Yamaguchi M (2020). Low tongue strength and the number of teeth present are associated with cognitive decline in older Japanese dental outpatients: a cross-sectional study. Int J Environ Res Public Health.

[11] Nagatani M, Tanaka T, Son BK (2023). Oral frailty as a risk factor for mild cognitive impairment in community-dwelling older adults: Kashiwa study. Exp Gerontol.

[12] Iwai K, Azuma T, Yonenaga T (2024). Longitudinal association of oral functions and dementia in Japanese older adults. Sci Rep.

[13] Peduzzi P, Concato J, Kemper E, Holford TR, Feinstein AR (1996). A simulation study of the number of events per variable in logistic regression analysis. J Clin Epidemiol.

[14] Fujiwara Y, Suzuki H, Yasunaga M (2010). Brief screening tool for mild cognitive impairment in older Japanese: validation of the Japanese version of the Montreal cognitive assessment. Geriatr Gerontol Int.

[15] Minakuchi S, Tsuga K, Ikebe K (2018). Oral hypofunction in the older population: position paper of the Japanese Society of Gerodontology in 2016. Gerodontology.

[16] Shimizu T, Ueda T, Sakurai K (2007). New method for evaluation of tongue-coating status. J Oral Rehabil.

[17] Kugimiya Y, Watanabe Y, Shirobe M (2021). A comparison of colorimetric and visual methods for the assessment of masticatory performance with color-changeable chewing gum in older persons. J Dent Sci.

[18] Tanaka T, Hirano H, Ikebe K (2023). Oral frailty five-item checklist to predict adverse health outcomes in community-dwelling older adults: a Kashiwa cohort study. Geriatr Gerontol Int.

[19] Kikutani T, Tamura F, Nishiwaki K (2009). Oral motor function and masticatory performance in the community-dwelling elderly. Odontology.

[20] Utanohara Y, Hayashi R, Yoshikawa M, Yoshida M, Tsuga K, Akagawa Y (2008). Standard values of maximum tongue pressure taken using newly developed disposable tongue pressure measurement device. Dysphagia.

[21] Kim EK, Lee SK, Choi YH (2017). Relationship between chewing ability and cognitive impairment in the rural elderly. Arch Gerontol Geriatr.

